# One-Shot Drilling Analysis of Stack CFRP/UNS A92024 Bonding by Adhesive

**DOI:** 10.3390/ma12010160

**Published:** 2019-01-06

**Authors:** Fermin Bañon, Alejandro Sambruno, Sergio Fernandez-Vidal, Severo Raul Fernandez-Vidal

**Affiliations:** Faculty of Engineering, Mechanical Engineering and Industrial Design Department, University of Cadiz, Av. Universidad de Cadiz 10, E-11519 Puerto Real, Cadiz, Spain; fermin.banon@uca.es (F.B.); alejandro.sambruno@uca.es (A.S.); sergio.fernandezvidal@mail.uca.es (S.F.-V.)

**Keywords:** adhesive, machining, modelling, dry, CFRP/UNS A92024

## Abstract

The use of adhesive layers can improve the properties and reduce the defects produced in the interfaces. This provides adherence to the structure, adapting the joining surfaces and avoiding spaces between the layers. However, the presence of the adhesive can potentiate the defects caused during drilling. In turn, a loss of adhesive in the interface can occur during machining affecting the final structure. This work has followed a conventional OSD strategy in CFRP and UNS A92024 aluminium sheet stacking with adhesive. A series of dry drilling tests have been developed with different cutting conditions and new noncoated WC-Co helical cutting tools. Analysis of Variance (ANOVA) statistical analyses and surface response models have been applied to determine the mechanical behaviour in the holes. For this purpose, the dimensional deviation, surface quality, and adhesive loss in the interface in relation to the number of holes have been considered. A combination of cutting parameters that minimizes the evaluated defects has been found. Diametric deviations and surface qualities below 2% and 3.5 µm have been measured in the materials that make up the stack with cutting speeds higher than 140 m/min and feed rates between 200 and 250 mm/min. However, the greatest adhesive losses occur at high cutting speeds.

## 1. Introduction

Today’s industry is looking for a production system that approaches optimum sustainability. Industrial sectors are looking for processes that present a balanced performance in energy, economic, social, functional, and environmental aspects [1,2].

The aeronautical sector is particularly noteworthy, being a benchmark in research, development, and innovation. One of the first challenges it faces in this fourth revolution is the automation of its processes. This is especially significant in operations such relevant as the assembly ones are to this industry, which involve extensive use of manual work. For this reason, the drilling operation is considered a key process due to the high number of holes made in an aircraft [3,4] for assembly through the riveting process.

These tasks must maintain the quality of the drilled holes avoiding the later elements separation. To achieve this goal, the drilling process is opting for drilling strategies known as OSD (One Shoot Drilling) [5,6,7]. The aim is to make a hole in a single step, regardless of the number of plates and the type of material. All this must satisfy the quality requirements imposed by the sector and avoid the use of lubricants.

Among the varying combinations of different materials that have to be joined together to form the aircraft, one of the most common is the union of composite material such as CFRP (carbon fiber reinforced polymer) and sheets of light metals such as aluminium alloys. This structure combines the strength of the fibres and the formability of the metal alloy [8,9,10].

Although mechanical joints are the most commonly used in the aeronautical sector, adhesive joints have also been widely used as a complement to riveting [11]. The existence of a layer that offers continuity between the materials that constitute the stack can offer a series of advantages. These benefits can be the elimination of defects in the interfaces between them, weight reduction, increased fatigue life of the joints, and a wide adaptability to materials [12,13,14]. However, the effect of cutting parameters on the adhesive has not been evaluated in previous studies. The presence of a third material in the interface of the hybrid structure may increase the defects generated during machining.

This paper proposes a study of the cutting parameters in the drilling of hybrid structures composed of CFRP and alloy UNS A92024, joined by an adhesive. The current objective is to drill hybrid structures in a single step. For this reason, the study of the influence of the cutting parameters on the final quality is essential and its influence on the final quality of the adhesive [15]. Defects such as an increase in the roughness of the composite material due to metal chip evacuation [16] or diameter variation may appear [17]. In addition, the inclusion of a third material as an adhesive can generate new defects in the interface of the materials producing a nonhomogeneous hole that meets the requirements established by the aeronautical sector. For the treatment of the results, Analysis of Variance (ANOVA) analysis and response surface models have been applied, which have been widely used in various studies on this topic [18,19,20,21,22]. Finally, a proposal is presented for the evaluation of the defectology caused in the adhesive that joins both materials.

## 2. Materials and Methods

For the development of this research, a WC-Co helical model drill without coating has been used. This was selected considering the materials that make up the stacks to be drilled, their thicknesses, the required qualities, and the cutting conditions. The dimensions and main characteristics are shown in Table 1. It has a double angle point, the section closest to the center corresponds to the highest point angle (140°) and the projection of the outermost edges provides a narrower angle (118°).

The material selected consisted of a hybrid structure presented in 210 mm × 210 mm sheets and composed of two different materials. It is formed by a 2 mm thick CFRP sheet and an 8 mm thick UNS A92024 alloy sheet. Specifications for both materials are shown in Table 2 and Table 3.

A two-component adhesive specific for structural elements was used to obtain the hybrid structure. It was applied by means of a hydraulic press in order to apply a constant pressure and guarantee a uniform thickness of 1 mm. The properties of the adhesive used are shown in Table 4.

**Figure 1:**
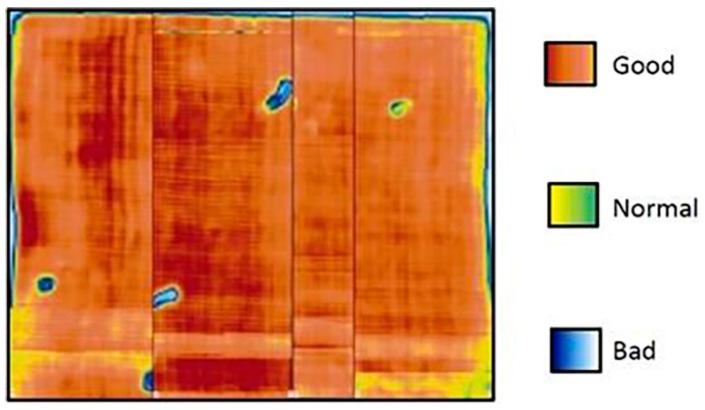
Ultrasound color map acquired on the hybrid stack interface for evaluating the adhesive application quality.

The adhesive was cured at room temperature in an air conditioned room. The polymerization temperature was 23 °C. After polymerization, an ultrasonic inspection was carried out to verify the correct bond between the materials. In Figure 1, the quality of the joint has been shown. It can be seen how the majority of the surface presents a continuous union. However, there are small defects in the edges of the stack, as well as air bubbles generated during the joining process.

**Figure 2:**
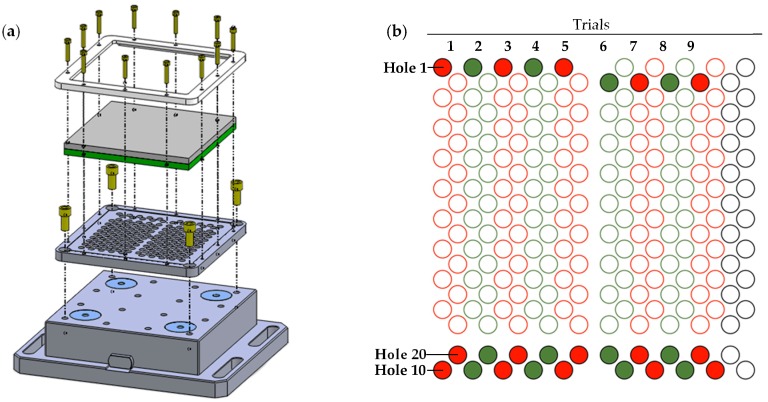
(**a**) Set up schematic representation. (**b**) Drill distribution for each trial.

The tests were carried out dry on a Kondia Five 400 5-axis machining centre (Elgoibar, Guipuzcoa, Spain), controlled by a Heidenhain iTNC530 control system (Traunreut, Bavaria, Germany). Three cutting speeds and forward speeds were combined to obtain a combination of nine tests (Figure 2). The set values for the cutting parameters have been defined on the basis of other studies and real application cases, and are indicated in Table 5.

**Figure 3:**
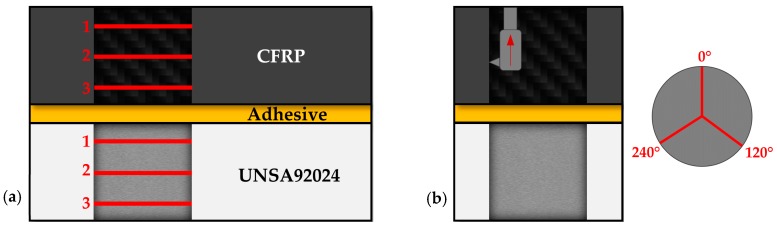
Evaluation methodology for (**a**) diameter and (**b**) quality surface.

Temperature has been measured using pyrometric techniques. Macro- and microgeometric defects were evaluated after machining. Diameters were evaluated using an inside micrometer of three contacts at three different heights per material (Figure 3a). On the other hand, the arithmetic mean roughness was evaluated by means of a rugosimeter using a cutoff of 0.8 mm to establish a comparison between the results obtained. Surface quality was evaluated in 3 generatrices per drill (Figure 3b).

**Figure 4:**
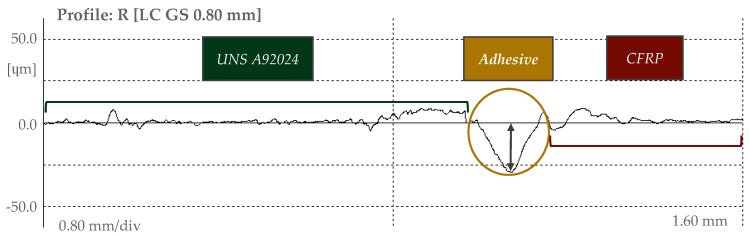
Evaluation of adhesive loss in the stack interface.

Finally, through the obtained roughness profiles, the loss of the adhesive in the interface of the materials was evaluated by means of image processing software, Figure 4. For each profile obtained the maximum depth of adhesive removed has been evaluated.

In order to assess the influence of input parameters on the results obtained a statistical analysis has been carried out. An ANOVA analysis through a Response Surface Methodology (RSM) has been implemented. With this technique an empirical model will be obtained that establishes a multiple linear regression.

## 3. Results and Discussion

The results obtained in the evaluation of the macro and microgeometric defects are shown in Table 6. The mean values and their respective deviations are shown prior to discussion.

### 3.1. Surface Quality

**Figure 5:**
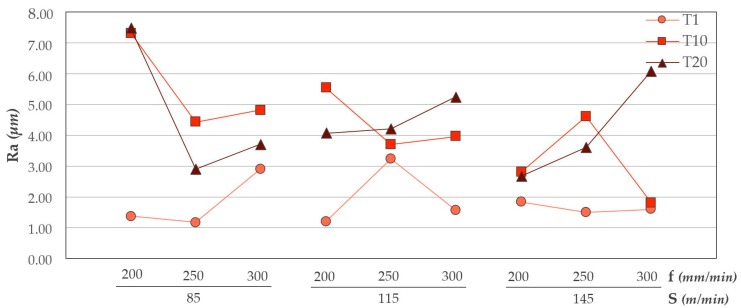
Ra values in CFRP for all trials carried out.

Cutting parameters are essential to obtain a correct surface quality. Figure 5 shows the values of Ra corresponding to the surface quality generated in the composite material. 

It shows how the quality deteriorates as the tool increases the number of holes. This can be related to wear geometry, both abrasive and adhesive, generated in the tool. As the number of holes increases, the machining capacity decreases, resulting in a worse surface quality.

It seems that the combination of a cutting speed of 145 m/min with a feed speed of 200 mm/min produces the least variation in the geometry used. For this combination the variation of Ra in the 20 holes is the lowest.

**Figure 6:**
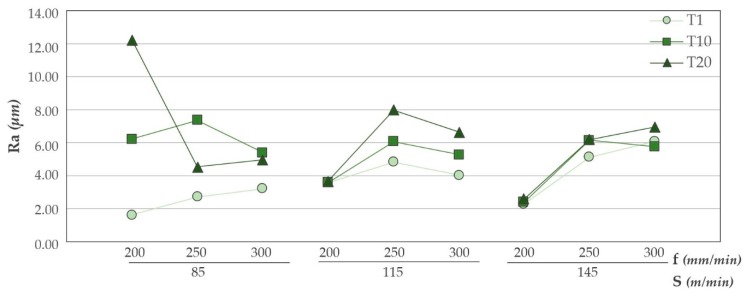
Ra values in UNS A92024 for all trials carried out.

Results obtained for the aluminium alloy are shown in Figure 6. It is appreciated how the feed rate significantly influences the surface quality obtained. The use of a feed rate of 200 mm/min generates the lowest Ra values. This may be because a smaller chip is generated, as Uddin et al. [23] indicate in their results. Increases in feed rate result in poorer surface quality. These results are consistent with those obtained here and shown in [3,4,15].

**Figure 7:**
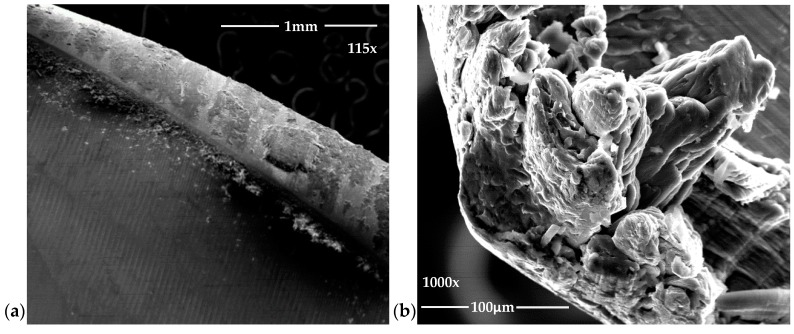
Adhesive wear generates in (**a**) the cutting edge and (**b**) the intersection of primary and secondary edges.

There is a clear dispersion of data as the tool achieves a greater number of machined holes. However, bigger dispersion between the first and the last hole is obtained for a cutting speed of 145 m/min. As it is known, increasing the cutting speed raise the drilling temperature, which can cause greater adhesion of the adhesive on the tool (Figure 7) providing a more homogeneous surface quality.

**Figure 8:**
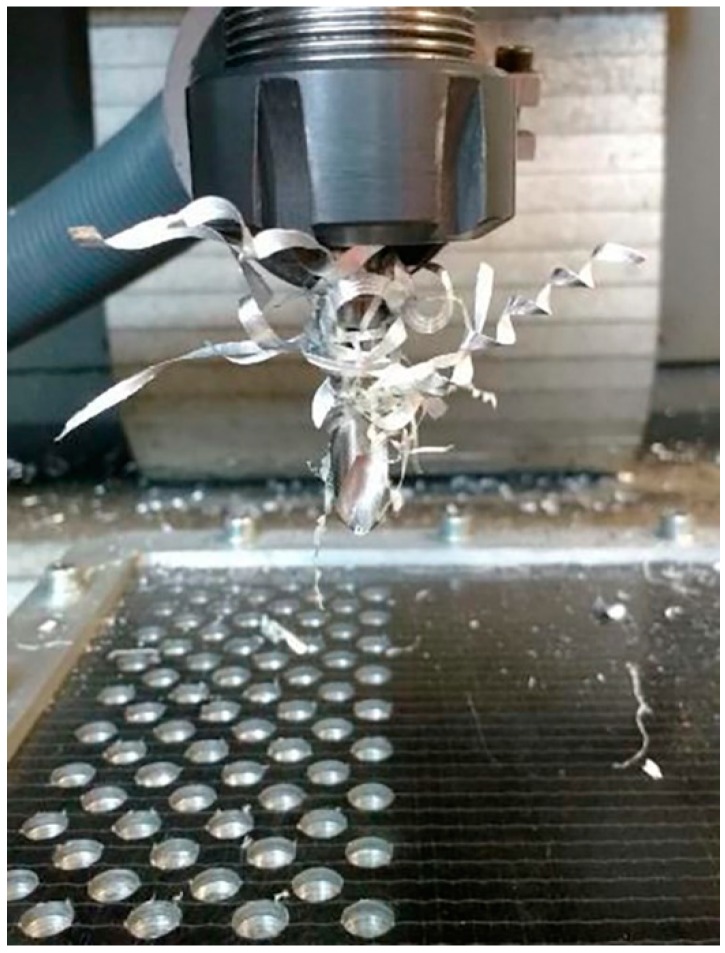
Adhesive and aluminium alloy chips adhered to the cutting geometry.

The appreciated values of Ra in both materials are within the values established by the aeronautical sector. At the same time, the data obtained experimentally present a great dispersion in all the combinations used. This may be due to the chips generated during machining (Figure 8). The presence of the adhesive on the interface seems to exacerbate the mechanism of adhesion of the aluminium alloy on the tool. This produces long thick chips adhered to the tool that do not come off at the end of the drill. As a result, when the next hole is drilled, this chip machines the composite material and part of the aluminum alloy until it is detached. This results in an uneven surface quality and very high Ra values.

This could be avoided by using a different drilling strategy such as vibration-assisted drilling [24,25]. By using this technique, the tool fragments the chip, reducing its size and facilitating its evacuation. In this way, a better surface quality could be obtained.

ANOVA statistical analysis has been carried out in order to identify the most influential parameters in the drilling of both materials (Table 7).

The number of holes drilled is the most influential variable as it has a *p*-value of less than 0.05 in the CFRP drilling, indicating that it is a statistically significant variable.

This is in line with what it was previously said about the progressive wear generated in the tool. Nevertheless, there is no apparent influence of the cutting parameters on the quality obtained.

Similar trends are shown in the results of the drilling of UNS A92024. The number of holes drilled is the most influential variable in surface quality. There is a dispersion for the values of Ra obtained with the increase in the number of holes. On the other hand, there is no influence of the cutting parameters used in the process.

From the results obtained, a contour diagram has been generated relating two variables. The contour diagrams are obtained from (1) and (2) with a R^2^ of 60.31% and 64.05%, respectively. The average error rate for the obtained values is 34.93% for CFRP material. On the other hand, the mean error rate for the aluminium obtained values is 22.85%. This high variation is again due to the formation of long chips adhered to the tool. The chips hit the surface of both materials producing a very random surface quality. Due to this, the model obtained is not able to follow the trends obtained experimentally.

Since the number of holes is the most influential variable, both the cutting speed and the feed speed for the two materials have been confronted with it.

**Figure 9:**
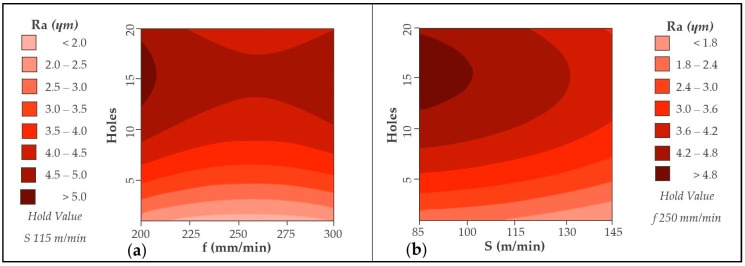
Contour diagram obtained by Ra values in CFRP: (**a**) Holes vs. feed rate and (**b**) holes vs. cutting speed.

CFRP drilling diagrams are shown in Figure 9. For both graphs the wear is progressive, generating an increase in Ra values. However, the use of high cutting speeds, close to 145 m/min softens this trend. This may be because the amount of material removed per turn is greater, resulting in a smoother and more homogeneous surface.

Ra(CFRP) = 22.2 − 0.071*S − 0.126*f + 0.492*Holes − 0.000171*S^2^ + 0.000161*f^2^ − 0.01412*Holes^2^ + 0.000382*S*f − 0.00028*S*Holes − 0.000103*f*Holes, (1)

Ra (UNS A92024) = 3.3 − 0.229*S + 0.077*f + 0.889*Holes + 0.000023*S^2^ − 0.000341*f^2^ − 0.00514*Holes^2^ + 0.001003*S*f − 0.00345*S*Holes − 0.001018*f*Holes, (2)

In turn, by increasing the amount of material removed, the temperatures reached are lower, without damaging the matrix. This would help to obtain a smoother surface.

**Figure 10:**
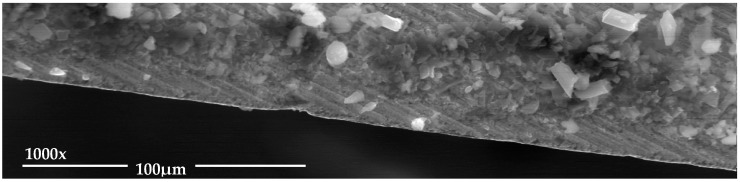
Abrasive wear generates in cutting edge.

On the other hand, the forward speed for a fixed cutting speed does not seem to be a very influential factor. The variation of Ra is very similar in all combinations. It can be seen how an intermediate feed rate, close to 250 mm/min, generates the smoothest trend while a speed of 200 mm/min can generate the highest Ra values. Elevated temperatures are related to forward speed reductions. This can lead to deterioration in the matrix, resulting in a more irregular surface. At the same time, it can generate greater friction between tool and material, increasing abrasive wear (Figure 10) and generating greater variation in results.

**Figure 11:**
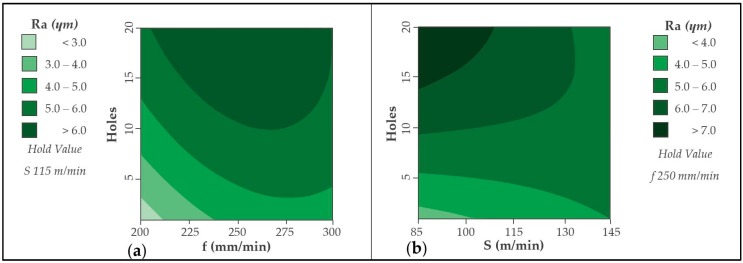
Contour diagram obtained by Ra values in UNS A92024: (**a**) Holes vs. feed rate and (**b**) holes vs. cutting speed.

Similar trends are shown in the diagrams corresponding to the drilling of the aluminium alloy (Figure 11). The selection of a high cutting speed, close to 145 m/min, together with a low feed speed, close to 200 mm/min, produces the best surface quality for both materials. This selection produces a smaller chip that is easier to evacuate and can reach lower temperature values. From a wear point of view, as the number of holes increases, there is a smoother tendency for this combination.

**Figure 12:**
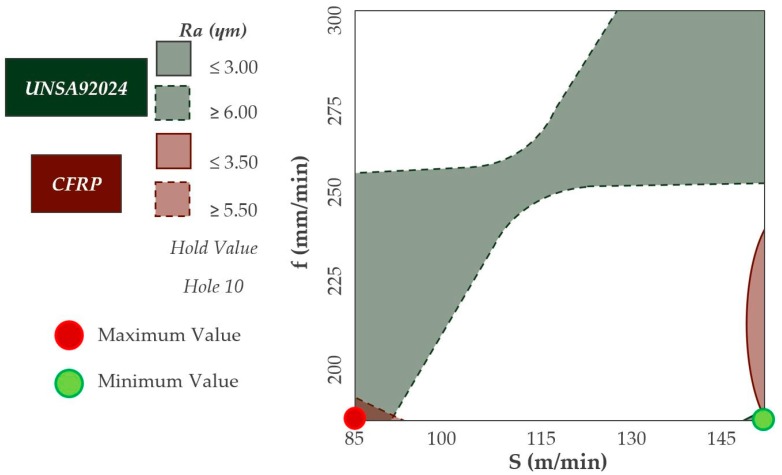
Overlap contour diagram by Ra values for CFRP & UNS A92024.

Two contour diagrams have been overlaid to determine the cutting parameters that minimize the values of Ra (Figure 12). The cutting speed and feedrate variables have been confronted. 

There is a small region generated by the combination of a cutting speed greater than 140 m/min and a feed rate close to 200 mm/min where the values of Ra are minimized. In this region it is possible to obtain values of Ra lower than 3 µm in the aluminium alloy and 3.5 µm for the composite material.

In addition, the selection of this combination is the one that offers a lower increase of Ra values by increasing the number of holes drilled as previously shown.

### 3.2. Diameters

**Figure 13:**
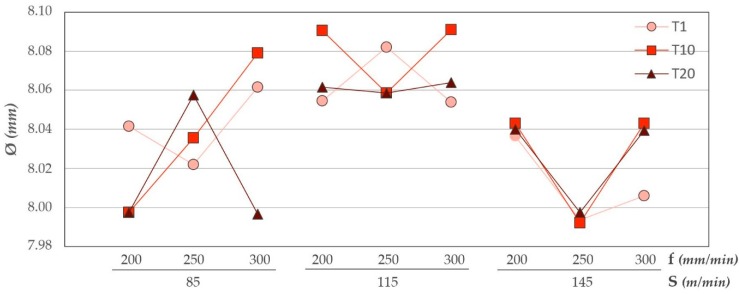
Diameter values in CFRP for all trials carried out.

**Figure 14:**
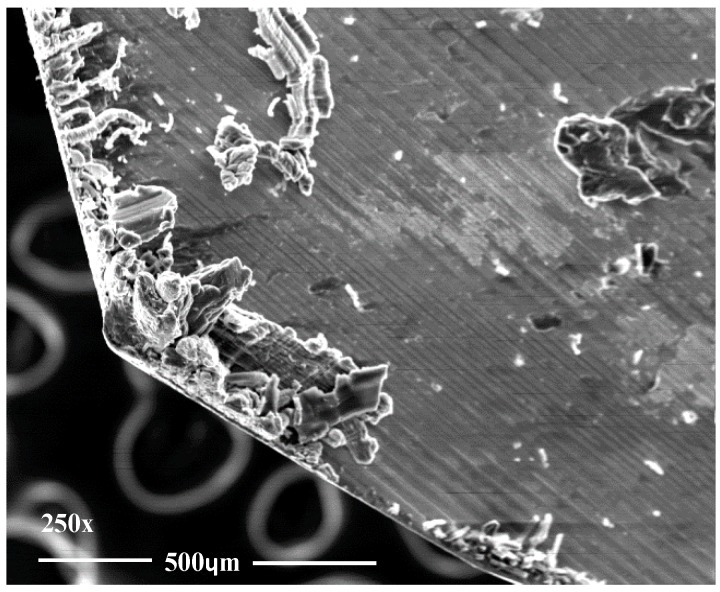
Adhesive wear generates in cutting edges.

Diameters evaluated in CFRP are shown in Figure 13. The number of drills performed does not seem to affect the diameters obtained in comparison with the values of Ra. The variation in results may be due to the adhesion and subsequent detachment of the aluminium alloy (Figure 14). This phenomenon modifies the geometry of the tool by varying its diameter.

However, an increase in the cutting speed results in closer values. This may be due to the increase of temperature in the cutting edges that would present a lower resistance in the matrix, facilitating its elimination. This is in line with the results obtained in the ANOVA analysis (Table 5). The cutting speed has the smallest *p*-value, being the most statistically influential variable.

Due to the very small thickness of the composite material, these diameter variations may also be related to the loss of adhesive in the interface. This loss could affect the measurement at a height close to the interface.

**Figure 15:**
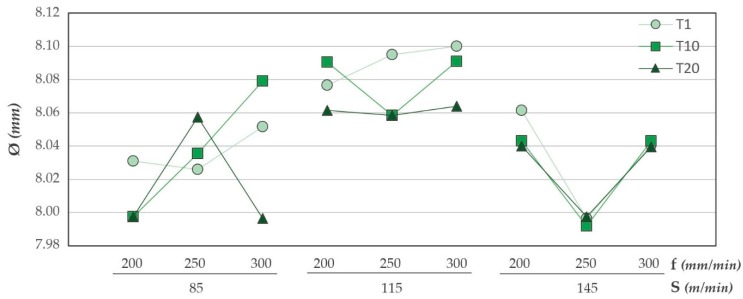
Diameter values in UNS A92024 for all trials carried out.

Diameters measured in aluminum alloy are shown in Figure 15. The results shown are very close to those obtained in CFRP. This may be due to the high adhesion of the material in the tool caused by process temperatures. A minimal dispersion is seen again when it comes to high cutting speeds. The increase of temperatures in the cutting zone can soften the material, facilitating its elimination and generating very uniform diameters.

No variable appears to be statistically significant in the ANOVA analysis performed for drilling aluminium alloy (Table 8). The adhesion and subsequent detachment of the material can produce random results. This produces that no input variable presents a high influence on the diameters obtained. However, the number of holes drilled is the variable with the lowest *p*-value. This means that within the randomness described, the number of holes machined is the most influential factor in the aluminium diameters. Therefore, the diameters generated in the aluminum alloy are related to the wear mechanisms produced. Adhesive type wear is predominant.

**Figure 16:**
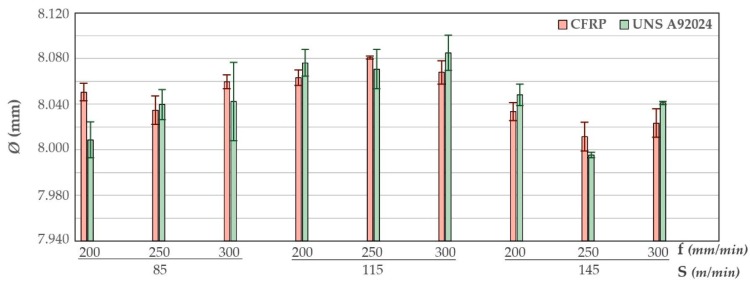
Diameter average values comparison for all cutting parameter combinations.

The average diameters and their respective deviations for the nine tests performed are shown in the (Figure 16). The diameters obtained in both materials are very close to each other except for Test 1. This is reflected in the ratio obtained between the diameters measured in CFRP and those measured in the aluminium alloy. The ratios obtained are between 0.998 and 1.005. This indicates that, independently of the cutting parameters, the diameters obtained are homogeneous and constant in the drill made.

This is consistent with the results given by D’Orazio. The temperature peaks are higher in the drilling of the composite material due to the low heat transfer efficiency. This, together with the friction of the metal chip when it is evacuated, produces larger diameters very close to those obtained in the aluminum alloy.

**Figure 17:**
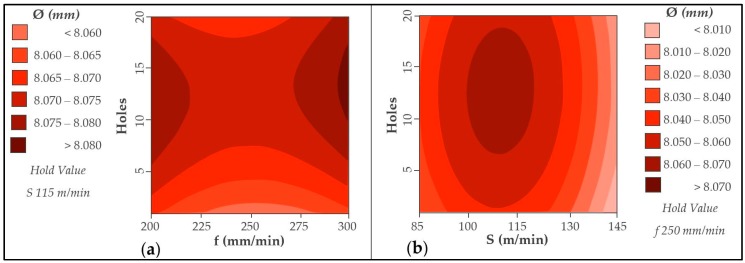
Contour diagram obtained by diameter values in CFRP: (**a**) Holes vs. feed rate and (**b**) holes vs. cutting speed.

The contour diagrams obtained are shown in Figure 17. The contour diagrams are obtained from the (3) and (4) with a R^2^ of 80.97% and 66.69%, respectively. The average error rate for the obtained values is 0.104% for CFRP material. On the other hand, the mean error rate for the obtained aluminium values is 0.180%.

Contrary to what is stated in the literature, an increase in the number of holes drilled results in an increase in the diameters obtained in CFRP for a fixed value of cutting speed. The adhesion of the metal alloy together with the adhesive itself may be enhanced by the temperatures reached during machining. This, together with the adhesion of the chip itself, can lead to an increase in the diameters obtained.

Ø(CFRP) = 7.695 + 0.00927*S − 0.00113*f + 0.00112*Holes − 0.000039*S2 + 0.000003*f2 − 0.000111*Holes2 − 0.000003*S*f + 0.000006*S*Holes + 0.000004*f*Holes,(3)

Ø(UNS A92024) = 7.524 + 0.01384*S − 0.00206*f − 0.00036*Holes − 0.000053*S2 + 0.000006*f2 − 0.000037*Holes2 − 0.000007*S*f + 0.000011*S*Holes − 0.000004*f*Holes,(4)

On the other hand, it should be noted that the use of low and intermediate cutting speeds affects the diameters obtained to a greater extent. The use of a speed close to 145 m/min, on the contrary, generates a diameter with few variations.

**Figure 18:**
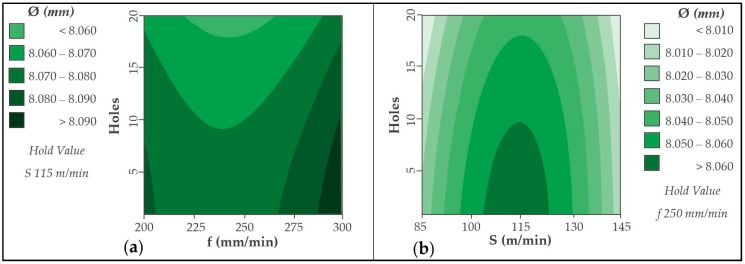
Contour diagram obtained by diameters values in UNS A92024: (**a**) Holes vs. feed rate and (**b**) holes vs. cutting speed.

The diameters measured in the aluminium alloy are reduced by increasing the number of holes drilled (Figure 18). For this material, it is observed that the intermediate cutting speed has a greater influence, achieving the highest values. Applying a low speed, 85 m/min, or high speed, 145 m/min, generates the smallest diameters of the stack.

**Figure 19:**
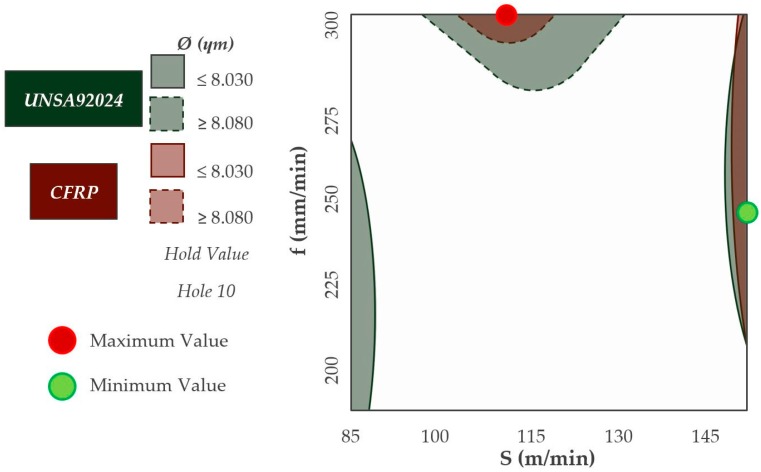
Overlap Contour diagram by diameter values for CFRP & UNS A92024.

The combination of cutting parameters that offer the most homogeneous diameter in both materials is shown in (Figure 19). Diameters very close to the nominal diameter are obtained by using cutting speeds higher than 140 m/min in both materials. However, the feedrate required to obtain these results must be close to 250 mm/min.

### 3.3. Adhesive

**Figure 20:**
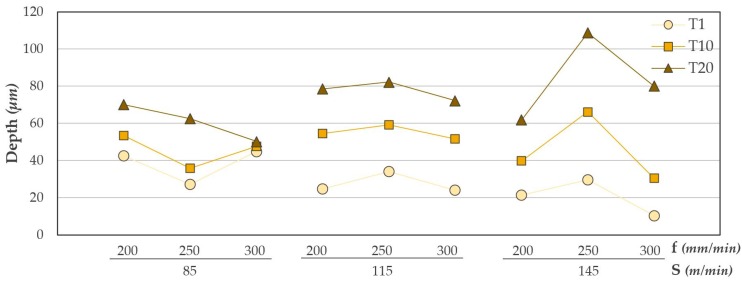
Adhesive loss values for all trials carried out.

An essential aspect is the final state of the adhesive after machining. The adhesive can be negatively affected and not perform its function. Adhesive loss in the interface can be influenced by the correct selection of cutting parameters. The maximum depth of adhesive lost after machining is shown in Figure 20.

**Figure 21:**
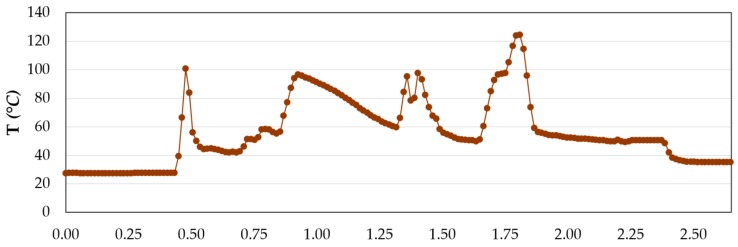
Temperature obtained for a single hole drilled.

This defect may be due to temperatures generated during machining (Figure 21). These may exceed the glass transition temperature of the adhesive itself. When this point is overreach, the adhesive softens and burns, facilitating its elimination by the cutting edges of the tool.

As with the results obtained in diameters and Ra, the number of holes drilled greatly affects the final quality of the adhesive: The amount of adhesive removed increases due to the progressive wear of the tool together with the adhesion of metal shavings. 

The amount of adhesive removed is also related to the cutting speed. This defect increases as the cutting speed values increase for a fixed feed value. This is consistent with the results obtained by L. Sorrentino [26]. As the cutting speed increases, the friction between the cutting edges and the surface of the material becomes greater. This produces an increase in the thermal energy generated, increasing the process temperatures. In the results obtained by L. Sorrentino, temperature peaks close to 180 °C are reached when drilling CFRP.

These temperatures exceed the glass transition temperature of the adhesive used (86 °C). At this point the material is softened and facilitates its removal at the interface. The results obtained at a speed of 145 m/min are particularly noteworthy. The observed tendency is inverse to the diameters evaluated. This would make sense, as the diameters close to the interface would increase due to the loss of adhesive. The ANOVA analysis carried out reflects the above statement (Table 9). The most influential variables in adhesive loss are the number of holes drilled and their combination with the cutting speed used. The advance speed, on the other hand, does not seem to have an apparent influence.

**Figure 22:**
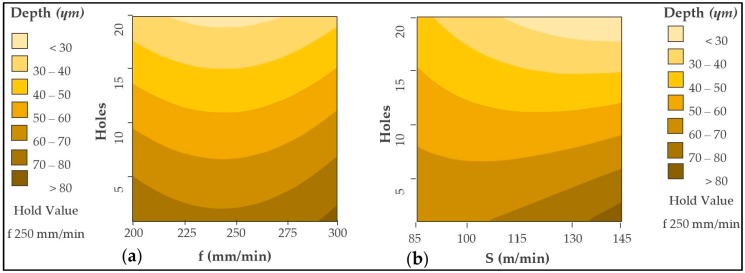
Contour diagram obtained by adhesive loss values: (**a**) Holes vs. feed rate and (**b**) holes vs. cutting speed.

The contour diagrams corresponding to the adhesive loss are shown in Figure 22. The contour diagrams are obtained from Equation (5) with a R^2^ of 82.99%. The average error value obtained was 18.25%. Although the speed of advance is not a very influential factor, it is appreciated that values close to 300 mm/min generate the minimum amount of adhesive eliminated in the first holes.

Depth (Adhesive) = −156 + 0.48*S + 1.512*f − 2.13*Holes − 0.00487*S^2^ − 0.00339*f^2^ + 0.0165*Holes^2^ + 0.00121*S*f + 0.0354*S*Holes + 0.00035*f*Holes, (5)

On the contrary, as opposed to the above for the values of diameters and Ra, a cutting speed close to 85 m/min should be selected. This combination of cutting parameters generates less adhesive loss in the interface as the number of holes increases.

## 4. Conclusions

In this work a methodology has been established for the analysis of dry drilling of adhesive bonded CRFP/UNS A92024 hybrid structures. The analysis of the results has been developed by diametric deviations, surface quality and adhesive loss through ANOVA techniques and the development of response surface models.

It has been determined that the surface quality is influenced by the cutting parameters. In addition, the loss of adhesive in the interface is related to the number of holes drilled.

The lowest values of Ra in both materials have been obtained by combining a cutting speed of 145 m/min with a feed speed of 200 mm/min. This can be produced by the smaller amount of material evacuated by the rotation of the tool, stabilizing the process temperatures.

The diametric deviations produced in both materials are very close. No variable has an influence on the diameters obtained by the ANOVA analysis. This is probably due to the randomness of the results obtained as consequence of secondary adhesion wear. This kind of wear affects the geometry of the tool with the adhesion and detachment of machined material, as a result of the stresses and temperatures generated during the process. The diametric ratios of both materials have always been near to 1. It has been seen that diameters very close to the nominal in both materials can be obtained by using cutting speeds higher than 140 m/min and feed speeds close to 250 mm/min.

Adhesive loss is directly related to increased cutting speed. This is due to the increase in temperature as consequence of the increased friction between the cutting edges and the machined materials. It has been determined that the cutting speed is the most influential factor in the process. Cutting speeds close to 85 m/min are recommended to reduce this defect. The feed rate does not have an excessive influence.

## Figures and Tables

**Table 1:** Drilling bit geometry.

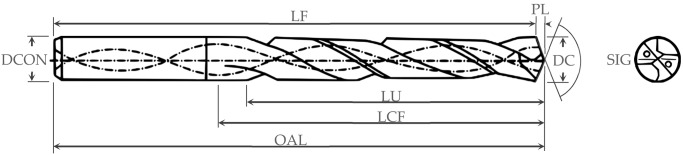									
DC (mm)	LU (mm)	DCON (mm)	SIG (°)	PL (mm)	OAL (mm)	LF (mm)	LCF (mm)	Helix Angle (°)	Material
7.92	25.00	8.00	118.00 140.00	1.20	80.00	78.80	30.00	29.82	Carbidre Substrate Uncoated

**Table 2:** Properties and features of CFRP.

Orientation (°)	Reinforcement	Matrix	Tensile Strength (MPa)	ILSS (MPa)	Tg (°C)
0/90	DowAksa A42 carbon fiber 49 Vol %	Epoxy DOW Voraforce 5300 51 Vol %	871	52	121

**Table 3:** Properties and features of UNS A92024.

Composition	Density (g/cc)	Ultimate Tensile Strength (Mpa)	Yield Tensile Strength (Mpa)	Elongation at Break (%)	Modulus of Elasticity (GPa)
Al 90.7–94.7%, Cu 3.8–4.9%, Mg 1.2–1.8%, Mn 0.30–0.90%, Zn 0.25%, Ti 0.15%, other 0.15%	2.78	425	310	≥10	73.1

**Table 4:** Characteristics of SAF30-LOT adhesive.

Polymerization	Operating Temperature (°C)	Tg (°C)	Elongation at Break (%)	Fixture Time (min)	Full Cure (h)	Lap Shear Strength (MPa)	Tensile Strength at Break (MPa)	Tensile Modulus (MPa)
Room Temp.	−40/150	86	50	120	24	16	8–10	180

**Table 5:** Combination of cutting parameters used for each test.

Trial	S (m/min)	f (mm/min)	Holes Machined ^1^	Lubrication
1	85	200	20	Dry
2	85	250	20	Dry
3	85	300	20	Dry
4	115	200	20	Dry
5	115	250	20	Dry
6	115	300	20	Dry
7	145	200	20	Dry
8	145	250	20	Dry
9	145	300	20	Dry

^1^ Holes 1, 10, and 20 have been analyzed.

**Table 6:** Result values of each experimental parameters.

Trial	Hole	Ø [mm (±µm)]		Ra [µm (±µm)]		Depth [µm (±µm)]
		CFRP	Al	CFRP	Al	Adhesive
1	1	8.031 (±0.816)	8.042 (±0.408)	1.38 (±0.033)	1.62 (±0.065)	42.584 (±0.781)
	10	7.998 (±1.225)	8.050 (±0.816)	7.32 (±0.033)	6.23 (±0.041)	27.136 (±0.887)
	20	7.998 (±0.408)	8.060 (±0.002)	7.47 (±0.024)	12.22 (±0.139)	44.910 (±0.678)
2	1	8.026 (±0.816)	8.022 (±1.633)	1.18 (±0.065)	2.71 (±0.163)	24.730 (±0.906)
	10	8.036 (±2.041)	8.052 (±1.225)	4.43 (±0.065)	7.35 (±0.163)	33.924 (±0.746)
	20	8.057 (±0.408)	8.031 (±0.408)	2.90 (±0.098)	4.53 (±0.122)	23.937 (±0.737)
3	1	8.052 (±0.408)	8.062 (±1.225)	2.92 (±0.049)	3.20 (±0.114)	21.322 (±0.431)
	10	8.079 (±0.816)	8.066 (±1.225)	4.82 (±0.057)	5.40 (±0.220)	29.644 (±0.707)
	20	7.997 (±1.225)	8.052 (±1.225)	3.72 (±0.147)	4.97 (±0.147)	10.351 (±0.687)
4	1	8.077 (±1.225)	8.055 (±1.225)	1.20 (±0.122)	3.58 (±0.147)	53.363 (±0.296)
	10	8.091 (±0.408)	8.071 (±0.816)	5.53 (±0.008)	3.57 (±0.204)	35.714 (±0.485)
	20	8.062 (±1.225)	8.064 (±1.633)	4.06 (±0.147)	3.68 (±0.131)	47.633 (±0.892)
	1	8.095 (±2.449)	8.082 (±1.633)	3.23 (±0.016)	4.82 (±0.057)	54.542 (±0.532)
5	10	8.059 (±0.408)	8.079 (±0.816)	3.71 (±0.114)	6.06 (±0.122)	59.167 (±0.558)
	20	8.059 (±1.289)	8.081 (±0.816)	4.21 (±0.041)	7.98 (±0.090)	51.512 (±0.401)
	1	8.100 (±1.633)	8.054 (±2.449)	1.57 (±0.024)	4.04 (±0.073)	39.640 (±0.719)
6	10	8.091 (±0.816)	8.071 (±0.408)	3.96 (±0.220)	5.28 (±0.139)	66.190 (±0.441)
	20	8.064 (±0.816)	8.079 (±0.816)	5.25 (±0.106)	6.66 (±0.033)	30.546 (±0.552)
7	1	8.062 (±1.225)	8.037 (±0.408)	1.84 (±0.090)	2.28 (±0.049)	70.171 (±0.474)
	10	8.043 (±0.816)	8.041 (±0.816)	2.81 (±0.057)	2.38 (±0.163)	62.413 (±0.550)
	20	8.040 (±1.633)	8.023 (±0.408)	2.67 (±0.155)	2.58 (±0.090)	50.236 (±0.575)
8	1	7.997 (±1.225)	7.994 (±1.225)	1.50 (±0.016)	5.12 (±0.090)	78.464 (±0.495)
	10	7.992 (±1.633)	8.020 (±2.449)	4.61 (±0.024)	6.15 (±0.139)	82.222 (±0.603)
	20	7.998 (±0.408)	8.021 (±0.816)	3.61 (±0.180)	6.19 (±0.057)	72.189 (±0.612)
9	1	8.041 (±0.408)	8.006 (±1.633)	1.59 (±0.016)	6.09 (±0.171)	61.680 (±0.457)
	10	8.043 (±0.816)	8.035 (±1.225)	1.82 (±0.057)	5.76 (±0.163)	108.765 (±0.257)
	20	8.040 (±1.225)	8.030 (±0.408)	6.08 (±0.106)	6.94 (±0.057)	80.120 (±0.596)

**Table 7:** Cutting parameters influence in Ra values by Analysis of Variance (ANOVA) analysis in both materials.

Source	DF	Adj SS	Adj MS	F-Value	*P*-Value
*CFRP*					
S (m/min)	1	5.1554	5.1554	2.65	0.122
f (mm/min)	1	0.3655	0.3655	0.19	0.670
Drills	1	30.8374	30.8374	15.88	0.001
Error	17	33.0218	1.9425		
Total	26	83.1892			
*UNS A92024*					
S (m/min)	1	1.416	1.4164	0.53	0.478
f (mm/min)	1	5.606	5.6057	2.08	0.167
Drills	1	27.602	27.6024	10.26	0.005
Error	17	45.741	2.6906		
Total	26	127.240			

**Table 8:** Cutting parameters influence in diameter values by ANOVA analysis in both materials.

Source	DF	Adj SS	Adj MS	F-Value	*P*-Value
*CFRP*					
S (m/min)	01	0.002912	0.002912	17.56	0.001
f (mm/min)	01	0.000008	0.000008	0.05	0.834
Drills	01	0.000425	0.000425	2.57	0.128
Error	17	0.002818	0.000166		
Total	26	0.014807			
*UNS A92024*					
S (m/min)	01	0.000017	0.000017	0.03	0.863
f (mm/min)	01	0.000616	0.000616	1.11	0.307
Drills	01	0.001531	0.001531	2.76	0.115
Error	17	0.009441	0.000555		
Total	26	0.028340			

**Table 9:** Cutting parameters influence in adhesive loss values by ANOVA analysis.

Source	DF	Adj SS	Adj MS	F-Value	*P*-Value
*ADHESIVE*					
Model	9	11168.5	1240.94	9.22	0.000
S (m/min)	1	16.6	16.6	0.12	0.730
f (mm/min	1	68.1	68.06	0.51	0.487
Drills	1	9235.4	9235.4	68.60	0.000
S (m/min) *Drills	1	1225.5	1225.47	9.10	0.008
Error	17	2288.6	134.62		
Total	26	13457.1			
